# The Significance of Plant Nutrition in the Creation of the Intestinal Microbiota—Prevention of Chronic Diseases: A Narrative Review

**DOI:** 10.3390/medicina60121969

**Published:** 2024-11-29

**Authors:** Miljana Z. Jovandaric, Kristina Jovanović, Misela Raus, Sandra Babic, Tamara Igic, Boba Kotlica, Srboljub Milicevic

**Affiliations:** 1 Department of Neonatology, Clinic for Gynecology and Obstetrics, University Clinical Center of Serbia, 11000 Belgrade, Serbia; 2 Department of Neurology, University Children’s Hospital, 11000 Belgrade, Serbia; 3 Department of Neonatology, University Children’s Hospital, 11000 Belgrade, Serbia; 4 Faculty of Medicine, University of Belgrade, 11000 Belgrade, Serbia; 5 Department of Gynecology and Obstetrics, Clinic for Gynecology and Obstetrics, University Clinical Center of Serbia, 11000 Belgrade, Serbia

**Keywords:** dysbiosis, intestinal microbiota, plant-based nutrition, diseases

## Abstract

Dysbiosis of the gastrointestinal tract is the most common cause of disease in childhood and adulthood. The formation of the intestinal microbiome begins in utero, and composition modification during life depends mainly on various genetic, nutritional, and environmental factors. The main cause of intestinal dysbiosis is improper nutrition due to a short period of breastfeeding, insufficient intake of fresh fruits and vegetables, and/or consumption of a large amount of processed food. The benefits of a diet based on grains, legumes, fruits, and vegetables are reflected in reducing the risk of cancer, cardiovascular diseases, myocardial infarction, stroke, rheumatoid arthritis, high blood pressure, asthma, allergies, and kidney stones. Anaerobic fermentation of fibers produces short-chain fatty acids (SCFA) that have an anti-inflammatory role and great importance in shaping the intestinal microbiota. Factors associated with high fiber in a plant-based diet promote increased insulin sensitivity. Insulin and insulin-like growth factor 1 (IGF-I) act as promoters of most normal and pre-neoplastic tissues. Conclusion: A plant-based diet high in fiber prevents disease by creating metabolites in the gut that reduce oxidative stress.

## 1. Introduction

Most people believe that bacteria in the body are the cause of illness or the development of certain diseases. The bacteria that make up the microbiome have a very important role in maintaining a strong and efficient immune system that participates in maintaining health [1].

In addition to the fact that bacteria influence the strengthening of immunity, they also influence the maintenance of the function of the digestive system, the proper function of the brain, and the balancing of hormone levels. Each of us has an internal complex ecosystem of bacteria that reside in our bodies that we call the microbiome. The microbiome is defined as a “community of microbes”. The vast majority of bacterial species that make up our microbiome live in our digestive system [2].

The human microbiome counts about 3.8 × 10^13^ microorganisms, which means that the ratio of bacterial cells to human cells is 1:1. More than 500 bacterial species are present in the intestines alone, which are also the most densely populated organ with microorganisms. The number and variety of these bacteria differ between individual parts of the intestine. The most important factors that affect the diversity of bacteria in the intestinal tract are pH, peristalsis, the presence of nutrients, and the amount of immunoglobulins in mucus. Consequently, the largest number of bacteria is present in the large intestine, due to the neutral pH and slow passage of contents through the lumen of the large intestine [3].

The present microorganisms achieve a symbiotic relationship with the host and have several vital functions. The most important role is protection against colonization by pathogenic bacteria. They achieve this effect by the production of bacteriocins and competition for nutrients, but also by stimulation of the host’s innate immunity. The intestinal microbiota has a metabolic role that is realized through the fermentation of indigestible carbohydrates. It also affects the absorption of water and electrolytes, the secretion of hormones, and the activation of the immune system [4].

During life, the formation of the microbiome occurs through the type of food that is consumed, daily exposure to bacteria, the amount of sleep, and the degree of stress [5].

Maintaining a strong immune system depends on the proper functioning of the digestive tract and a balanced level of hormones that affect brain function [6].

The human microbiome differs from the place in which the organisms are located. Different microorganisms live in different parts of the body, prefer different foods, and perform various functions. There is an oral microbiome; a skin microbiome, which has many subcategories (e.g., armpit, nose, foot, etc.); and a genital microbiome, as well as a gut microbiome. Our microbiomes are called our “genetic fingerprint” because they help determine our DNA and show the tendency to developing diseases, as well as influence hereditary factors. It is thought that 90 percent of all diseases can be traced in some way to the composition of the gut microbiome. For a long time, it was considered that the microbiota are acquired during and after childbirth, but recent research supports the acquisition of microbiota in utero and connects it with the microbiota of the placenta and uterus [7].

Considering all the roles played by the gastrointestinal microbiota, during its dysregulation, i.e., the occurrence of dysbiosis, all its roles are changed, which leads to immune dysfunction and the development of many diseases in early childhood and adulthood. Dysbiosis is associated with the emergence of irritable bowel syndrome, inflammatory bowel diseases, asthma, obesity, neurological diseases, and many others [8].

In addition, nutrition in the prenatal and postnatal periods are the most important factors for the predisposition and manifestation of diseases in the adult period [9].

New research suggests that reducing meat intake can have significant benefits for the gut microbiome. The gut microbiome plays a key role in various aspects of human health, including digestion, immune function, and metabolism. Individuals who followed a vegetarian or vegan diet had a more diverse and beneficial composition of gut bacteria compared to those who consumed a diet rich in animal products [10].

This greater diversity of gut microbes is associated with improved gut health and a reduced risk of various chronic diseases. Furthermore, plant-based diets usually contain more fiber, which acts as a prebiotic, providing food for beneficial gut bacteria. As we continue to explore the intricate relationship between diet and gut health, it is becoming evident that reducing our meat intake and embracing plant-based alternatives can have profound effects on our overall well-being, highlighting the need to rethink our relationship with food in the context of veganism [11].

### The Microbiome of a Newborn

A newborn’s microbiome represents the sum of the genetic information of microorganisms that are present not only in the newborn’s intestinal tract but also on the skin of the newborn and its mother. The formation of the microbiome composition of the newborn’s gut occurs during pregnancy (depending on the lifestyle of the pregnant woman, geographic origin, race, disease, nutrition, and length of gestation) [12].

The most important moment in the formation of the microbiome is intrauterine, i.e., (fetal and metabolic) programming, which participates in the development of tissues and organ systems and begins with the formation of the embryo and later the fetus. The first changes in the microbiome of the fetus occur intrauterine, and at the time of birth, further development and changes in the composition of the microbiota continue [13]. It has been proven that the microbiome plays a very important role in the development of the digestive system, as well as neurodevelopment during the first years of life [14]. A newborn’s microbiome changes so that the predominance of anaerobes consisting of *Escherichia coli* and *Streptococcus* are replaced by anaerobes consisting of *Bifidobacterium*, *Bacteroides*, and *Clostridium* [15].

The microbiome affects health by regulating the immune system via the gut–brain axis. It has been proven that indole-3-lactic acid (ILA) produced by bifidobacteria can convert the immunoregulatory galectin-1 in Th2 and Th17 cells, thus regulating the immunity of the newborn [16]. Animal data suggest that the administration of *Lactobacillus reuteri* could reverse social deficits in autism spectrum disorder (ASD) models by rescuing synaptic plasticity via the vagus nerve [17].

In premature children, the presence of *Klebsiella* affects the increased number of T cells and reduced secretion of neuroprotectors, which results in brain damage in premature children. Even short-term changes in the composition of the intestinal microbiome during the first weeks of life can have a minimal impact on immune cells and an effect on neurophysiology at an early stage of development [18].

Newborns who were born vaginally are colonized by the mother’s vaginal flora, in which lactobacilli are the most common *Escherichia* and *Bacteroides*, while babies born by cesarean section are colonized by bacteria found on the surface of the mother’s skin and in the operating room, dominated by *Staphylococcus*, *Enterococcus*, and *Klebsiella* [19].

Therefore, they are more prone to metabolic and immunological diseases, as well as neurodevelopmental disorders [20].

As for antibiotics, if the mother takes antibiotics during pregnancy, it represents a potential risk for the development of autism [21]. For a long time, it was believed that the fetus has a sterile gastrointestinal tract; however, it has been proven that intestinal colonization begins before birth. The composition of the microbiome of a pregnant woman’s gut, vagina, skin, and breast milk affects the colonization of newborns. Although the function of the placental microbiome is not clear, it is known that the gut microbiota during pregnancy is the most important factor in the health of the offspring. Bacteria from the intestine of a pregnant woman reach the amniotic fluid, the placenta, and also the milk ducts of the breast [22].

**Figure 1:**
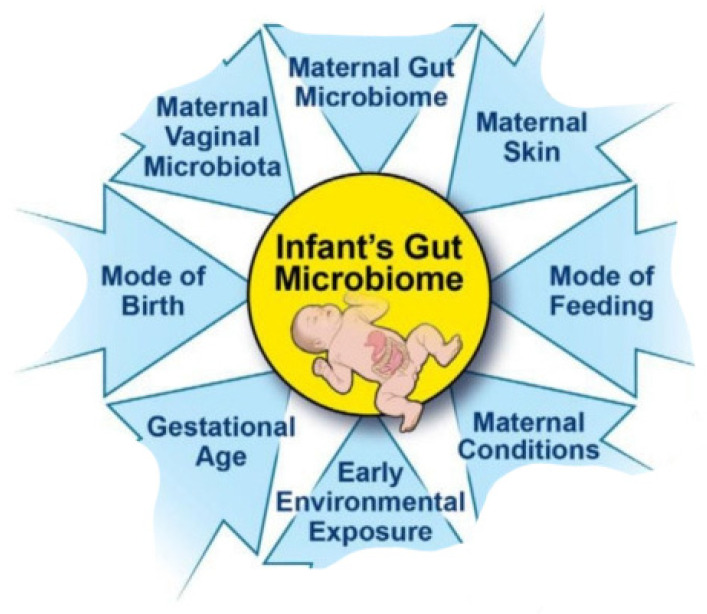
Factors affecting the microbiome of a newborn.

Immediately after birth, facultative anaerobic bacteria colonize the digestive tract. Over time, the facultative anaerobic bacteria in the intestine of a newborn are replaced by obligate anaerobes because the digestive tract becomes more anaerobic with the transition to solid food. Transmission after birth occurs between people living together, and transmission is affected by the number of people, proximity, frequency of social interactions, resistance to colonization, etc. The transmission of these bacteria is very important for the human microbiome because it enables their spread and maintenance in the population [23] (Figure 1).

The formation of the newborn’s microbiome is influenced by many factors, such as the way of birth, nutrition in the first months of life, premature birth, maternal illnesses before pregnancy, the condition of the pregnant woman, smoking, stress, the use of antibiotics, length of hospitalization, as well as environmental factors.

## 2. Nutrition and the Microbiome

It is generally known that the organism is inhabited by microorganisms that are included in the normal flora. However, only in the 21st century, with the development of molecular biology and metagenomics, was its enormous diversity and importance for health revealed. It is a collection of bacteria, archaea, protozoa, fungi, and viruses that inhabit the skin and mucous membranes. Normal flora received a new name—microbiota and the genetic material of all microorganisms—the microbiome. The number of bacterial cells is ten times greater than the number of cells in our body, and bacterial DNA contains three hundred times more genes that encode proteins [24]. The composition of the microbiota in each person varies throughout life. In early childhood and old age, it differs from some of the characteristics of middle age [25]. The first time when the composition of the microbiome is most similar to an adult is around three years of age [26].

The microbiome changes with age due to various influences, where, in addition to genetics, the environment and diet play a very important role in the composition of the microbiome, such that in adults the bacteria of the genera *Firmicutes* (*Lachnospiraceae* and *Ruminococcaceae*), *Bacteroidetes* (*Bacteroidaceae*, *Prevotellaceae*, and *Rikenellaceae*), and *Actinobacteria* (*Bifidobacteriaceae* and *Coriobacteriaceae*) dominate the microbiota [27]. The composition and quantity of the microbiota varies along the digestive tract, such that in the intestines it is the most numerous, with a predominance of anaerobic bacteria of about 90% in the large intestine, of which the most abundant are *Bifidobacterium*, *Bacteroides*, *Eubacterium*, *Ruminococcus*, *Faecalibacterium*, and *Blautia* [28].

One gram of feces contains 10^11^ bacteria, and 30% of the fecal mass consists of microbiota. The microbiome consists of the core microbiome, which consists of the types of microorganisms present in more than 95% of people (but in varying numbers), and the secondary microbiome, originating from a small number of different species that differ among individuals. Research has established that each person has a “personal” microbiome [29]. In addition to the way of birth, the core microbiota is influenced by the individual’s genetic and immunological factors, as well as the way of eating and personal hygiene. Examining the intestinal microbiota of African people who eat mainly plant-based foods and the populations of highly developed countries where more meat and fast food are consumed, a large difference in the profile of bacteria in the intestines was determined [30].

Additionally, the composition of the microbiota is affected by exposure to xenobiotics, i.e., various foreign chemical substances, especially pesticides. Xenobiotics can have a toxic, immunogenic, or carcinogenic effect on our body, and they additionally disturb the normal microbiota [31]. Glyphosate is mostly used as a herbicide in countries of low socioeconomic status. It achieves its effect by inhibiting the synthesis of plant-derived amino acids such as tryptophan, tyrosine, and phenylalanine, which it achieves through the inhibition of the enzyme 5-enolpyruvylshikimate-3-phosphate synthase (EPPSS) in the shikimate pathway of synthesis in plants, fungi, bacteria, protozoa and archaea, limiting the bioavailability of these amino acids for humans. In this way, the microbiome changes, because baby food formulas that contain a low level of glycosyl lead to a decrease in the number of Bifidobacteria, while an increase in Clostridium, Enterorhabdus, Parasutterella, Blautia, Escherichia, and Shigella was observed in adults [32].

During an individual’s lifetime, the microbiome changes due to eating habits, such that the intestinal flora is changed by antibiotics, a diet rich in sugar and refined carbohydrates, toxins—gluten and refined oils, stress, and age. One of the biggest imbalances in the intestinal flora is the modern diet. Foods that are rich in sugar and refined carbohydrates (e.g., bread, pasta, biscuits, and cakes) encourage the growth of fungi, especially Candida, which favors the colonization of parasites [33]. Certain studies have shown that artificial sweeteners such as aspartame can stimulate the growth of unhealthy bacteria in the gut microbiome, such as an increase in the total number of Enterobacteriaceae and Clostridium leptum. A high-fat diet leads to a decrease in Bacteroides and an increase in Firmicutes, while the presence of high-fat and aspartame has the opposite effect, reducing the increase in Firmicutes at the same time without affecting the level of Bacteroidetes [34].

To establish and maintain a balance of good and bad bacteria in the intestines, the diet must contain fruits and vegetables rich in plant fibers, then whole grains, legumes, nuts, and lean sources of animal origin, such as fish, chicken, and turkey. A diet with a variety of foods, but in their natural form, makes the gut microbiome the most optimal. In particular, one should include legumes, such as beans, but also fruits in their menu, because these foods contain a lot of plant fiber and can promote the growth of good bacteria in the intestines [35]. A diet dominated by fruits and vegetables has shown a decrease in the relative abundance of bacteria from the family Lachnospiraceae, such as Ruminococcus and Lachnobacterium [36]. Fermented foods such as kefir, sauerkraut, kombucha, tempeh, miso, and sourdough bread, contain healthy bacteria and can reduce the number of bacterial species that can cause various diseases in the intestines. Consuming these foods changes the gut microbiome by increasing the concentration of Lactobacillus, Lactococcus, and Bifidobacterium, and decreasing the concentration of Proteobacteria and Enterobacteriaceae [37].

A diet composed of a variety of foods, in their natural form and grown organically, makes the gut microbiome diverse. Especially legumes, such as beans, but also fruits, contain a lot of plant fibers and can promote the growth of Bifidobacteria and Lactobacilli in the intestines [38]. Prebiotics are a type of fiber that encourages the growth of Bifidobacterium and Lactobacillus including Faecalibacterium prausnitzii and Akkermansia muciniphila. Foods rich in prebiotics include artichokes, bananas, asparagus, oats, and apples [39].

Polyphenols are plant compounds that have antioxidant, anti-inflammatory, and antibacterial effects, and are contained in fruits, vegetables, cereals, beans, tea, coffee, honey, red wine, olive oil, and whole grains. Caffeic acid is most often found in fruits and vegetables, while ferulic acid is found in cereals, especially rice and wheat bran. Garlic and onion are particularly rich in quercetin, while flavanols are found in red wine, chocolate, and lotus root. Anthocyanidins in plants mainly exist in glycosidic forms, which are usually called anthocyanins, and they are most abundant in strawberries, blueberries, and cherries. The group of polyphenols also includes capsaicinoids in chili peppers, avenanthramides from oats, and resveratrol, which is found in the red and purple skins of grapes and grape wine [40]. They are broken down by the gut microbiome and thus stimulate the growth of healthy bacteria. Polyphenols can selectively inhibit the growth of the pathogenic bacteria Clostridium, Ruminococcus, and Clostridium histolyticum and stimulate the growth of Bifidobacterium, Enterococcus, acid bacteria, Roseburia, Prevotella, and Lactobacillus [41].

Whole grains contain plant fibers such as beta-glucan, which bacteria from the gut break down, thus having a positive effect on the gut microbiome. Bifidobacterium and Bacteroides are the most important intestinal bacteria and make up 80% of the microbial space in the intestines, whose role is to break down glycans from food [42]. Beneficial bacteria in the gut participate in the production of vitamins K, B1, B2, B3, B6, and B12 and amino acids [43]. It also participates in the synthesis of short-chain fatty acids: butyrate, acetate, and propionate. These acids have extremely significant physiological effects. Butyrate is a source of energy for colon cells, has a beneficial effect on the intestinal barrier and the immune system, and has an anti-inflammatory effect. It prevents the multiplication and causes the death (apoptosis) of malignant cells. Acetate also has an anti-inflammatory and anti-cancer effect in the intestines, increases blood flow to the intestines, and can be a source of energy for muscles and the brain. Propionate lowers blood cholesterol levels, reduces fat formation in the liver, and reduces insulin resistance [44].

Intestinal flora plays a major role in the immune system. Over two-thirds of the body’s immune protection is located in the intestines, and the beneficial bacteria play a role in the activation of important parts of the immune system—lymphocytes and immunoglobulins that destroy viruses, bacteria, fungi, and parasites. Beneficial bacteria in the intestines are very important because they activate the immune system, neutralize toxins, and heavy metals, deactivate carcinogenic substances, and participate in the digestion and maintenance of the intestinal epithelium. The imbalance of intestinal flora weakens immunity and its natural protective food and defenses [45].

## 3. Plant-Based Nutrition

A diet based exclusively on foods of plant origin is called a vegan diet, which is based on grains, legumes, fruits, and vegetables. A subgroup of the vegan diet is a diet that includes the intake of only fresh, raw fruits and vegetables without thermal processing. Veganism is completely reduced to a plant-based diet, while vegetarians also consume products of animal origin, e.g., eggs, honey, milk, and milk products [46].

A plant-based diet has less cholesterol and fat than other types of diet. Its basis consists of vegetables, vegetable proteins, good fats, and whole grains. According to previous studies, vegans make up 0.2% to 1.3% of the American population and 0.25% to 7% of the world population [47].

The basis of plant nutrition is fiber, and retention in the intestines depends on their solubility in water, so they slow down digestion and give a feeling of satiety. This especially applies to the fibers found in fruits and legumes, which create viscous substances in water. Fibers that come from whole grains and vegetables are less soluble and therefore increase the amount of fecal mass, which enables regular emptying of the intestines [48]. Fiber is a type of carbohydrate that exists in three main forms: soluble, insoluble, and fermented fiber. Soluble fibers dissolve in water and slow down digestion. They can help reduce cholesterol and blood sugar levels in the body. Insoluble fibers do not dissolve in water and play a different role in digestion. They add bulk to our stool and pass through the system faster. They help the intestines work better and prevent constipation. Fermented fibers come from both categories and help increase healthy bacteria in the colon [49].

**Figure 2:**
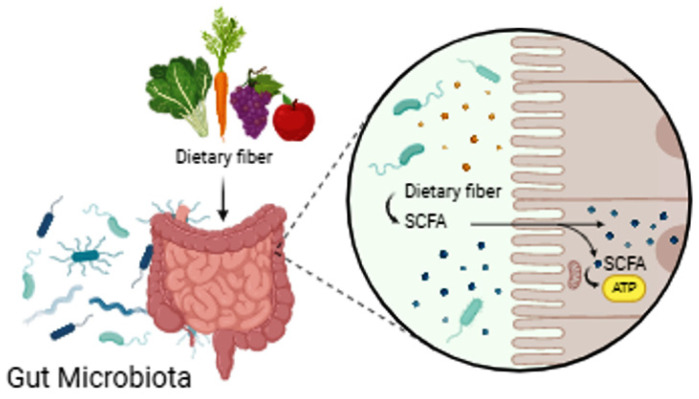
The importance of short-chain fatty acids (SCFA).

Carbohydrates of plant fibers are not digested by enzymes of the digestive tract, such as amylase, but by anaerobic fermentation in the intestinal lumen, which produces short-chain fatty acids (SCFA), such as acetate, propionate and butyrate, that participate in lipid metabolism, maintaining cholesterol and glucose levels, as well as in the immune response of the intestinal barrier by creating conditions for the growth of *Lactobacillus* and *Bifidobacteria* [50] (Figure 2).

Carbohydrates of vegetable fibers are digested by anaerobic fermentation in the lumen of the intestine, during which SCFAs that participate in lipid metabolism, maintaining cholesterol and glucose levels, as well as in the immune response of the intestinal barrier by creating conditions for the growth of Lactobacillus and Bifidobacteria. SCFAs are absorbed into the cells and lining of the colonic epithelial cells that use SCFAs for oxidation processes. Butyrate is mostly used as an energy source for colonic epithelial cells. Epithelial cells of the large intestine metabolize SCFAs, the end product of which is adenosine triphosphate (ATP), which is used for the metabolic needs of the cells. Numerous transmembrane proteins, receptors, and transporters that bind SCFAs and other monocarboxylic acids are found in a large number of cells, including neurons, so they regulate inflammation and carcinogenesis and maintain the function of the intestinal barrier.

Indigestible plant fibers and complex carbohydrates participating in the production of SCFAs lower the pH value in the colon, which has an impact on the microorganisms present and consequently on the production of SCFAs. Most SCFAs are absorbed in the proximal colon via active transport, which brings bicarbonate into the colon to neutralize the resulting acids [51].

It has been proven that bacteria of the genus *Bacteroidetes* stagnate in growth in conditions of lower pH, while bacteria of the genera Bacteroidetes and Firmicutes, which are among the largest producers of SCFAs in the intestines, show greater tolerance to lower lumen pH values. A lower pH has a role in protecting the organism from the excessive growth of pathogens that are sensitive to changes in the pH value [52].

In addition to pH, iron is necessary for the functioning of microorganisms and the production of SCFAs. Iron is absorbed in the duodenum, while unabsorbed iron enters the lumen of the large intestine. Almost all bacteria require iron for growth; although, members of the *Lactobacillus* genus are exceptions due to the use of iron alternatives such as manganese and cobalt. In addition, *Bacteroides* species are considered heme auxotrophs and therefore have an absolute requirement for heme (or inorganic iron in combination with protoporphyrin). Total levels of unabsorbed iron in the colon affect the composition and activity of the colonic microbial ecosystem. Such effects have been reported in both in vitro and in vivo studies, and common changes induced by inorganic iron supplementation include reductions in beneficial *Bifidobacterium* and *Lactobacillus* spp. and promotion of potentially harmful *Enterobacteriaceae* spp. [53]. Iron deficiency causes a decrease in butyrate production and gene expression for butyryl-CoA:acetate-CoA transferase, the enzyme that catalyzes the final step in butyrate synthesis [54].

The main energy source for colonocytes is SCFAs; about 60–70% of their total energy is generated by the oxidation of SCFAs. Absorbed SCFAs in the large intestine via the portal vein are transported to the liver, where they become precursors for other molecules such as glucose (propionate is a precursor for gluconeogenesis), ketones, and fatty acids. SCFAs that are not utilized in the liver for metabolic processes are transported to the heart, skeletal muscles, and brain, where they are an important source of energy [55].

SCFAs play an important role in maintaining health by inhibiting the production of cytokines, IL-12, TNF-α, and IL-1β, as well as by inhibiting the activity of the transcription factor nuclear factor-kappaB (NF-kappaB), which controls the expression of some inflammatory cytokines genes [56]. In addition, by regulating the function of neutrophils, macrophages, and dendritic cells, as well as the differentiation of T and B lymphocytes, it mediates the cellular immune response [57].

The epithelial layer of cells lining the intestinal tract is part of the innate immunity that recognizes pathogenic microorganisms and their metabolites, and intestinal epithelial cells (IECs) are a source of mucin, antimicrobial peptides, cytokines, and chemokines that are secreted into the intestinal lumen. Cytokines and chemokines are mediators of the immune response that regulate the activation of cells of the immune system [58].

A high concentration of the short-chain fatty acids that come into contact with IECs was found in the intestine. SCFAs are absorbed into cells by passive diffusion and active transport via monocarboxylate transporter associated with hydrogen ion (monocarboxylate transporter 1; MCT1) or monocarboxylate transporter 1 associated with sodium (SMCT1). IECs use butyrate as an energy source in the form of ATP, while propionate and acetate are transported to the liver. Butyrate plays a role in IEC homeostasis by stimulating colonocyte proliferation and apoptosis. In addition, it encourages exfoliation of cells and thereby contributes to the regeneration of intestinal cells [59].

Tight junction proteins or zonula occludens proteins (tight junction; TJ) are proteins found at the junction of endothelial and epithelial cells. They regulate the transport of solutes and signal molecules that promote cell differentiation and polarization. SCFAs indirectly affect TJ proteins by modulating cytokines. Interferon γ (IFNγ) and tumor necrosis factor α (TNF-α) increase the permeability of cell junctions via TJ proteins [60]. By suppressing inflammatory cytokines, SCFAs prevent cell permeability. By activating the GPR109A receptor, butyrate suppresses the increase in cell permeability [61].

SCFAs directly affect the formation of TJ proteins in epithelial cells; activation of GPR109A increases the expression of proteins claudin-3, occludin and zonula occludens 1, which are components of the tight intercellular junction. The formation of TJ proteins is most affected by metabolism, especially increased endogenous concentrations of calcium ions due to the activation of the enzyme AMP-activated protein kinase. Butyrate regulates the activity of this enzyme and thereby improves the function of the intestinal epithelial barrier [62,63].

The association between intestinal microbiota and SCFAs was established in a plant-based diet characterized by an increased amount of *Firmicutes* (around 59%) and *Bacteroidetes* (at 39%) compared to the total intestinal microbiota of vegans. Increased concentrations of acetate and propionate in vegans are due to intestinal fermentation of fiber by *Bacteroidetes* spp., while *Firmutes* are responsible for butyrate synthesis [64,65].

The gut microbiome interacts with the brain through the microbiota gut–brain axis, regulating various physiological processes that affect brain function neurally, humorally, and immunologically. The gut microbiota regulates the brain by the bacteria themselves, microbially derived metabolites (e.g., SCFAs, secondary bile acids, and amino acid metabolites), and bacterial cell wall components [66]. SCFAs affect the human body through the regulation of histone acetylation and methylation of G-protein coupled receptors (GPCRs), facilitating the secretion of various hormones (e.g., GLP-1 and PII) and neurochemicals (e.g., serotonin) and the induction of vagus nerve signaling [67].

**Figure 3:**
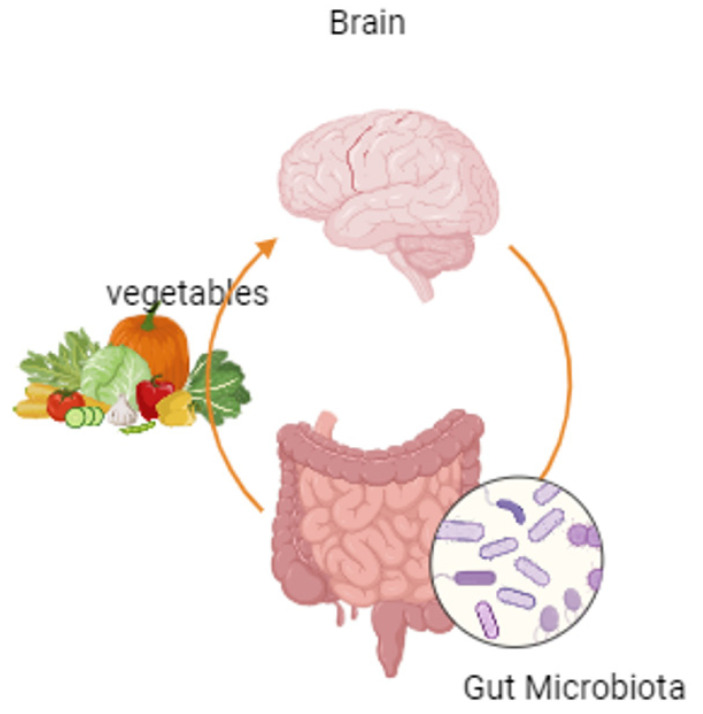
The microbiota gut–brain axis.

SCFAs are also involved in the functions of gastrointestinal functionality, blood pressure regulation, circadian rhythms, (neuro)immune function, emotional stability, and behavior, as butyrate has been shown to improve cognitive impairment in vascular dementia, and propionate can reduce reactions to high-energy food in the striatum. There is also an association between low SCFA levels in patients with depression, anxiety, and psychosocial stress [68] (Figure 3).

During the digestion of plant-based food, there is a connection between the gut and the brain. SCFAs are involved in gastrointestinal functions, blood pressure regulation, circadian rhythms, (neuro)immune functions, emotional stability, and behavior, as butyrate has been shown to improve cognitive impairment in vascular dementia, and propionate can reduce responses to high-energy foods in the striatum. There is also a link between low SCFA levels in patients with depression, anxiety, and psychosocial stress.

Dietary fibers increase sensitivity to insulin and improve overall metabolic health, reducing cardiovascular diseases, as well as the occurrence of cancer, improving cognitive functions, and thus inducing better physical and psychological tolerance to stress [69]. Higher fiber intake from cereals has been shown to result in a modulated inflammatory condition that is often associated with insulin resistance. The vegan diet also helps in reducing body weight and cholesterol levels, regulating blood glucose levels, and improving the function of the digestive tract [70]. The effect on glucose metabolism mediated through SCFAs is achieved by stimulating the secretion of glucagon-like peptide-1 (GLP-1) and PII-like signaling proteins, leading to an increase in insulin, reduced glucagon production, and improved sensitivity to insulin [71,72,73].

The effects of SCFAs in the liver prevent the process of glycolysis and gluconeogenesis, stimulating the formation of glycogen. In skeletal muscles, activation of AMP-activated protein kinase (AMPK) leads to increased expression of the GLUT4 glucose transporter, which improves glucose uptake in muscles [74]. SCFAs also have an impact on lipid metabolism, by stimulating the oxidation of fatty acid tissue, with a consequent effect on the change in concentration of total cholesterol [73,75].

The basis of all diseases is oxidative stress, which leads to damage to cellular structures, including membranes, lipids, proteins, lipoproteins, and cell DNA. Oxidative stress represents the metabolic state of the organism followed by an increase in the amount of reactive oxygen molecules. These are primarily the superoxide radical O_2_, hydrogen peroxide (H_2_O_2_), and the hydroxyl radical OH. An excess of hydroxyl radicals and peroxynitrite causes lipid peroxidation, with consequent damage to the cell membrane and lipoproteins. One of the main products of the oxidative degradation of lipids is malondialdehyde (MDA). Aldehydes and conjugated diene compounds formed by lipid peroxidation show cytotoxic and mutagenic properties [76].

An organism fights daily with various harmful substances to which it is exposed through food, water, air, or internal processes. Mitochondria take food and oxygen from the outside environment and make energy in the form of ATP, carbon dioxide, and water. During the normal process of cellular respiration in the mitochondria, free radicals are produced that can cause various damages to the body. In addition, canned and processed food, pesticides, insecticides, herbicides, polluted water and soil, heavy metals, UV rays and other electromagnetic radiation, chronic and severe infections, smoking, alcohol, and stress lead to increased production of free radicals [77].

Free radicals are highly unstable and reactive molecules that take electrons from the lipids of the cell membrane, which damages the cell membrane itself and disrupts the integrity of proteins and the structure and functionality of DNA, resulting in mutations. Elevated levels of reactive oxygen species (ROS) are a major cause of aging and age-related diseases, including Parkinson’s disease, diabetes, metabolic and cardiovascular diseases, and cancer [78]. In the fight against free radicals, acetate, propionate, and butyrate formed by the fermentation of plant fibers by intestinal microbiota play a key role. Butyrate is mostly used as an energy source for colonic epithelial cells. Epithelial cells of the large intestine metabolize SCFAs through beta-oxidation and the citric acid cycle (Krebs cycle), the end product of which is adenosine triphosphate (ATP), which is used for the metabolic needs of the cells [79]. Numerous transmembrane proteins, receptors, and transporters that bind SCFAs and other monocarboxylic acids are found in many cells, including neurons. Most studies have shown that SCFAs affect the regulation of inflammation, carcinogenesis, intestinal barrier function, and oxidative stress [80].

Diet affects about 30% of all cancers in developed countries and 20% in developing countries. Plant fiber bulks up stool, which reduces transit time and, in turn, may reduce colonic epithelial cell exposure time to potential fecal carcinogens. Second, bacteria in the gut can ferment fiber into short-chain fatty acids such as butyrate. These fatty acids can promote colon cell differentiation and normal cell apoptosis [81].

Insulin-like growth factor-I (IGF-I) is a peptide hormone synthesized in the liver, whose role is to stimulate the growth of cells in the body, It is regulated by diet, where food intake increases its production and it decreases abundance in states of starvation. Intake of a large amount of plant foods with a high fiber content in the diet promotes increased insulin sensitivity [82]. A plant-based diet is associated with lower circulating levels of total IGF-I and higher levels of insulin-like growth factor binding protein I (IGFBP-I) and IGFBP-2 compared to a meat-eating diet or even a lacto-ovo-vegetarian diet. Insulin and IGF-I act as promoters of most normal and pre-neoplastic tissues. Therefore, their reduction may reduce cancer rates [83,84].

It has been established that a diet rich in refined foods, full of simple sugars and trans fats, with poor nutritional composition and high energy value (caloric food), can cause the accumulation of bad bacteria in the intestines. This increases the risk of developing obesity, as well as numerous other health problems. To establish and maintain the balance of good and bad bacteria in the intestines, the diet must contain fruits and vegetables rich in plant fibers, then whole grains, legumes, nuts, and lean sources of animal origin, such as fish, chicken, and turkey. Plant foods contain very important micronutrients such as magnesium, calcium, and potassium, as well as bioactive elements such as polyphenols carotenoids, and antioxidants, which reduce the occurrence of chronic diseases by reducing oxidative stress and lipid peroxidation of cell membranes [85]. Carotenoids from plants have anti-carcinogenic, anti-inflammatory, and antimicrobial properties. The mechanism of action includes the elimination of free radicals by neutralization, blocking lipid peroxidation of cell membranes [86]. Polyphenols are natural bioactive plant compounds that have an antioxidant effect as a result of neutralizing ROS. In addition, polyphenols from black and green tea inhibit the growth of harmful bacteria such as *Staphylococcus aureus*, *Escherichia coli*, *Salmonella*, *Pseudomonas aeruginosa*, *Helicobacter pylori*, and *Listeria monocytogenes* [87].

Ferulic acid is hydroxycinnamic acid, which has strong antioxidant properties, can neutralize harmful free radicals, and also stimulates the production of endogenous antioxidant enzymes. In addition, it has good anti-inflammatory effects, such as the influence on the metabolism of arachidonic acid (inhibiting the synthesis of prostaglandin PEG2, etc.) and inhibiting mediators of inflammation (such as IL-1β, IL-6, TNF-α, etc.) and can also reduce the activation of mast cells. Pure ferulic acid, an isoferulic acid, can inhibit the production of interleukin-8 (IL-8) [88]. Anthocyanins belong to the group of flavonoids that are found in all plants and their parts. The bioflavonoids that have the strongest active factors (catechins from tea, hesperidin from citrus, resveratrol from red wine, rutin and quercetin from citrus, isoflavones, and pycnogenol) show strong antioxidant, anticarcinogenic, and anti-inflammatory action [89]. Vitamin C (ascorbic acid) acts as an antioxidant and protects the body from oxidative stress, and participates in the biosynthesis of collagen, L-carnitine, and certain neurotransmitters, as well as protein metabolism. Collagen is an essential component of connective tissue’s vital role in wound healing. In addition to its biosynthetic and antioxidant functions, vitamin C plays an important role in immune function and improves the absorption of non-heme iron, the form of iron present in foods of plant origin. Insufficient intake of vitamin C causes scurvy [90]. Vitamin E acts as an antioxidant. Its importance is reflected in the protection of cells from lipid peroxidation and the body’s defense against viruses, bacteria, and various diseases, which is thanks to the synergistic effect from vitamins C and D and zinc. It helps form red blood cells (erythrocytes), prevents blood clotting in blood vessels, and promotes vitamin K absorption [91,92].

### Disadvantages of Plant-Based Nutrition

A vegan diet can be extremely beneficial for health when properly planned and implemented, providing all the necessary nutrients and health benefits such as improved heart health and weight control [93]. This type of diet requires careful planning, especially when meeting the daily needs for protein, calcium, iron, and certain vitamins. Sources of protein in a vegan diet include legumes, tofu, nuts, seeds, and soy. For calcium, vegans rely on dark green leafy vegetables, almonds, calcium-fortified tofu, and fortified plant-based beverages. Iron can be found in legumes, nuts, seeds, and fortified cereals [94].

Due to the greater need for nutrients during the period of intensive growth, vegan children are affected by nutritional deficiencies and slow growth; their motor and cognitive development are the most endangered. Moreover, vitamin B12 deficiency in children leads to severe, long-lasting megaloblastic anemia and neurological disorders, including cognitive impairment and short-term memory loss [95]. In addition, children on a plant-based diet tend to be shorter and weigh less than the general population of their age [96]. A special concern for vegans is the lack of vitamins B12 and D, as well as minerals such as iron, calcium, and zinc. These nutrients are often present in animal products, so it is important to find plant-based alternatives or consider supplementation. Supplementation can be key, especially when it comes to vitamin B12, which is essential for the nervous system and red blood cell production. Vitamin B12 is not naturally found in plant foods, so supplementation or consumption of fortified foods is often necessary [97]. Some foods are fortified with vitamin B12, such as muesli, cornflakes, and soy drinks. Mushrooms and seaweed, which vegans often rely on, have trace amounts of vitamin B12. However, that form is either inactive or the bioavailable amount is insufficient. That is why it is recommended to take supplements at least occasionally. Vitamin B12 deficiency symptoms in vegans can be masked by taking folic acid. However, the possible consequences of demyelination of some types of nerves are very serious [98]. Low levels of vitamin B12 and omega-3 fatty acids are associated with increased levels of homocysteine and decreased good cholesterol in the blood. This increases the risk of atherosclerosis and cardiovascular disease [99].

This type of food is not recommended for pregnant women or breastfeeding mothers due to the increased need for nutrients and minerals [100]. The birth weight of fetuses and newborns of women on a vegan diet is lower compared to non-vegan mothers. Low birth weight is an unfavorable outcome, as it is a major contributor to the development of cardiovascular diseases later in life [101]. Inadequate nutrition during intrauterine life leads to functional and structural changes in the fetus, which consequently leads to various diseases, such as hypertension, diabetes, and obesity [102].

Childhood is a crucial period for establishing lifelong healthy eating habits. The environment can have a significant impact on these habits. The eating habits of children depend on geographical origin, the eating habits of their parents, their socioeconomic status, attendance at preschool institutions (kindergarten), and later influences at school (i.e., during the adolescent period children are influenced by the eating habits of their peers) [103].

Given that the number of people identifying as vegan has increased dramatically, the issue of vegetarian or vegan diets in children remains an unresolved controversy [104]. If the child’s diet is exclusively of plant origin, there is a deficit in the quantity and quality of protein, as well as iron, zinc, selenium, calcium, riboflavin, vitamins A, D, B12, and essential fatty acids, namely DHA or a-linolenic acid. In vegan children, vitamin D and iron levels are deficient due to insufficient intake or excess dietary fiber, which is rich in phytates and oxalates that reduce their bioavailability [99].

Considering the benefits of a vegan diet, teenagers and children who need more protein, iron, and minerals for growth should have a balanced diet that does not damage intestinal homeostasis [105].

In some diseases, such as celiac disease (CB), where in genetically predisposed individuals the immune system reacts to ingested gluten from cereals (wheat, rye, barley, oats) and leads to damage of the mucosa of the small intestine followed by chronic inflammation of the mucous membrane of the small intestine with the progressive disappearance of intestinal villi leading to decreased absorption of many nutrients, including iron, vitamin B12, folate, copper and zinc, a plant-based diet can be challenging [106].

## 4. Mediterranean Diet

Despite the numerous advantages of a plant-based diet, the best way of eating is the Mediterranean diet, which is associated with a high intake of plant-based foods, a reduced intake of saturated fats and processed carbohydrates, and a high intake of vegetable fibers [107].

The Mediterranean diet (MD) is characterized by a moderate intake of lean meat (rabbit, chicken, turkey) and fermented milk products, milk, and cheese; the use of fat in the form of olive oil; an abundance of fruits, vegetables, and cereals rich in dietary fiber and antioxidants; deep-sea blue fish such as mackerel, tuna, and salmon, which are rich in omega-3- polyunsaturated fats;, and nuts (walnuts) rich in monounsaturated fatty acids, as well as a moderate amount of red wine with a meal. The MD uses a lot of spices such as garlic, onion, and leek, which are rich in probiotics, as well as fresh or dried aromatic herbs (parsley, oregano, mint, rosemary, thyme, coriander, basil) and spices (cumin, cloves, saffron, cinnamon, pepper...), which act as antioxidants, but also have an anti-inflammatory effect [108].

It is generally known that omega-3 (n-3) polyunsaturated fatty acids (PUFAs), eicosapentaenoic acid (EPA), and docosahexaenoic (DHA) acid play an important role in maintaining health and preventing many diseases. The amount in the body depends on the type of diet. Thus, the MD contains an abundance of fatty fish as a key source of polyunsaturated fatty acids (PUFAs), which are known to improve the health of the cardiovascular system. In addition to fish and seafood, nuts are also a source of PUFAs within the MD. Among the PUFAs, ω-3 (derived mainly from fish oil) is known to reduce inflammatory processes, and ω-3 also helps prevent breast cancer development through the induction of reactive oxygen species-mediated macrophage death [109,110].

Consumption of flaxseed oil (a source of PUFAs in the form of α-linoleic acid) reduced *Firmicutes* and *Blautia* within the gut microbiota. Based on the association of Firmicutes and Blautia with inflammatory cytokines such as TNF-α, PUFAs improve the inflammatory response by modulating the gut microbiota. In addition to enhancing the immune–inflammatory status (as a precursor to many chronic non-infectious degenerative diseases), ω-3 PUFAs also improve the intestinal epithelial barrier, reducing its permeability in colitis [111,112]. The lower frequency of cancer, cardiovascular, autoimmune diseases, diabetes, and cardiovascular diseases is explained by the composition of the microbiota in people who adhere to the MD. Adherence to the MD influences microbiota profiles and SCFA production, with the microbial diversity of the MD being associated with an enrichment of Firmicutes and Bacteroidetes and an increase in fecal SCFAs [113].

The Western style of nutrition is opposite to the MD andis known for very high-calorie meals with a lot of fat, red meat, carbohydrates, refined sugars, and very few vegetables and fruits. The frequency of diseases in people who use fast food can be explained by microbiota with a predominance of *Ruminococcus* and *Streptococcus* bacteria and a reduced amount of SCFAs [114,115].

A well-balanced diet, with supplements in some periods of life, adapted to the individual’s age, activities, and health condition, is an important factor in preserving health and longevity [116] (Table 1).

## 5. Conclusions

A plant-based diet with plenty of fiber enables the formation of beneficial intestinal bacteria that regulate numerous cellular processes with their metabolites, and it has become evident that reducing meat intake and accepting plant alternatives can have profound effects on health.

## Figures and Tables

**Table 1:** Nutrition according to age and special needs of the organism.

Children Up to 12 Months of Life	Breastfeeding + Varied Diet + Supplements (Doctor’s Control)
Children up to 3 years of age	Varied diet + supplements (doctor’s control)
Children older than 3 years and adolescents	Varied diet (vegetable diet + supplements only under the supervision of a doctor)
20–30 years	Plant-based diet + supplements (nutritionist control) or varied diet
30–40 years	Plant-based diet + supplements (nutritionist control) or varied diet
40–60 years	Plant-based diet + supplements (nutritionist control) or varied diet
Over 60 years	Plant-based diet + supplements (nutritionist control) or varied diet
Pregnant women	Varied diet + supplements (under the control of a doctor)
Women who are breastfeeding	Varied diet + supplements (under the control of a doctor)
Special pathological conditions (diseases of the gastrointestinal tract, carcinoma, allergy to certain types of food)	Varied diet + supplements (under the control of a doctor)
Athletes	Plant-based diet + supplements (supervised by a nutritionist) or varied diet + supplements (under the supervision of a doctor)

## Data Availability

All data are available in the archives (databases) of Medline and PubMed.
